# Goal Attainment and Outcome Evaluation for Upper and Lower Limb Spasticity: A Real-Life Cohort Analysis in a Community-Based Outreach Service

**DOI:** 10.3390/toxins18090398

**Published:** 2026-09-18

**Authors:** Aideen Steed, Heather Williams, Jessie Alfonso, Denise Hancock, Stephen A. Ashford

**Affiliations:** 1 Regional Hyper-Acute Rehabilitation Unit, Northwick Park Hospital, London Northwest University Healthcare NHS Trust, London HA1 3UJ, UK; aideen.steed@nhs.net (A.S.); heather.williams4@nhs.net (H.W.); jessie.alfonso@nhs.net (J.A.); denise.hancock@nhs.net (D.H.); 2 The Hillingdon Hospital NHS Trust, London UB8 3NN, UK; 3 Department of Palliative Care, Policy and Rehabilitation, Cicely Saunders Institute, Florence Nightingale Faculty of Nursing, Midwifery and Palliative Care, King’s College London, London SE5 9PJ, UK

**Keywords:** active function, passive function, Arm Activity Measure (ArmA), Leg Activity Measure (LegA), spasticity, goal attainment scaling, botulinum toxin A, real-world evidence

## Abstract

Botulinum toxin type A (BoNT-A) is an established treatment for spasticity. Goal Attainment Scaling (GAS), alongside standardized measures such as Arm Activity Measure (ArmA) and Leg Activity Measure (LegA), enables evaluation of patient-centered outcomes. This real-world cohort study evaluated goal attainment and standardized measure functional changes following BoNT-A for arm and/or leg spasticity, evaluated using GAS as well as passive function subscales of ArmA (ArmA-PF) and LegA (LegA-PF), and active function subscales (ArmA-AF; LegA-AF). Inclusion in the analysis was based on documented baseline and post-intervention GAS scores following BoNT-A therapy (*n* = 390). Across 855 treatment cycles, 1258 goals were set and categorized, of which 150 (11.9%) were related to active function and 705 (56.0%) to passive function. The mean GAS T-score improved from 36.2 to 49.4 (change 13.1, *p* < 0.001), with significant gains observed for both passive (change 13.7, *p* < 0.001) and active goals (change 12.6, *p* < 0.001). Passive function improved significantly on ArmA-PF (change 2.5) and LegA-PF (change 2.1), while significant changes in ArmA-AF and LegA-AF were not seen. In conclusion, goal-directed spasticity management incorporating BoNT-A resulted in improvements in goal attainment, which corresponded to a clinically meaningful change in passive function measured by ArmA-PF and LegA-PF, but not for active function. Passive function was the most common treatment priority. Findings provide real-world evidence to support community-based spasticity services and the use of structured goal setting, ArmA and LegA application, in routine practice.

## 1. Introduction

Spasticity is a common feature of upper motor neuron syndrome following damage to the central nervous system, which commonly leads to reduced activity and function. Treatment can include repeated injections of Botulinum Toxin Type A (BoNT-A), physical therapy rehabilitation programs, and/or the use of oral antispasmodic medications for upper and lower limb spasticity [1]. BoNT-A is a safe, effective focal intervention for spasticity [2], with several BoNT-A products licensed for this purpose in the United Kingdom (UK) and is internationally recommended for use in routine clinical practice [3]. In addition to medical staff, UK legislation governing the prescribing and administration of medications enables physiotherapists, nurses, and, in some cases, other allied health professionals to be trained to inject and/or prescribe BoNT-A and other pharmacological agents used in the management of spasticity [4]. Repeated BoNT-A injections have been shown to deliver long-term benefits across multiple clinical issues for upper or lower limbs [5,6,7,8,9,10]. Techniques such as electro-stimulation, electromyography, and ultrasound guidance can be used to improve localization accuracy, treatment efficacy, and safety, with the potential for improved outcomes [11,12]. Clinical outcomes may also be influenced by differences in the BoNT-A formulation used, the dose of BoNT or physical intervention applied, as well as other anti-spasticity agents, the muscles treated, variability in clinical diagnosis, and the number of treatment cycles applied for the management of a specific goal of intervention [13,14,15,16].

Specialist Community Outreach Services, based at and integrated with specialized regional services, are part of the UK service specification, with the aim of supporting people with severe complex disability following an acquired brain injury in their journey from acute services to the community. They typically have a catchment area of 50-to-100 mile radius and a catchment population in excess of 1 million. The service in this case provides spasticity and related physical management through a mixture of home visits and satellite clinics to meet the needs of patients otherwise unable to access treatment, ensuring equity for this group.

Goal Attainment Scaling (GAS) is a well-established, structured approach for setting and evaluating goal achievement [17,18,19,20]. Evaluation of overall attainment can be calculated through a cumulative GAS-T score. In clinical practice or research, the GAS-T score is valuable when patients set multiple goals, with variable levels of achievement. The minimal clinically important difference (MCID), which reflects the smallest change perceived as clinically meaningful, has been established for spasticity management evaluation by GAS as a change score equal to or greater than 10 [21]. Treatment goals are agreed to target “active function” where improvement in voluntary motor control is anticipated, or “passive function”, where the primary aim is to facilitate care and prevent further complications (e.g., maintaining palmar hygiene), often when recovery of motor control is not expected [22]. GAS is a standardized evaluation used in most specialized services in the UK.

Studies examining arm spasticity management have demonstrated that person-centered goals can be categorized consistently into six main goal areas mapping to domains of the WHO International Classification of Functioning, disability and health (ICF), helping group treatment goals to specific domains [23,24]. Leg impairment and passive function categories are broadly similar to those identified in arm spasticity with an exception for active function, which is divided into transfers and walking for the leg [25]. ICF goal categorization has therefore been established as follows:

**Domain 1: symptoms/impairment**

- Pain/discomfort;
- Involuntary movement;
- Range of movement/contracture prevention.

**Domain 2: Activities/function**

- Passive function (ease of caring for the affected limb);
- Active Function (including mobility);
- Facilitating therapy.

This work has aided the development of the Focal Spasticity Index, a structured framework that combines individualized goal setting with standard outcome measures, facilitating more consistent evaluation and comparison of treatment outcomes (https://www.kcl.ac.uk/nmpc/assets/rehab/focal-spasticity-index-sa-2018.pdf, accessed on 10 September 2026).

Active and passive function are both important in the achievement of clinically relevant goals for patients undergoing spasticity management. The Arm Activity Measure (ArmA) [26] and Leg Activity Measure (LegA) [27] were developed specifically to provide comprehensive assessment of both active and passive function (as well as symptoms and quality of life) in the context of interventions for arm spasticity in the arm and leg. Both measures are patient- or carer-reported with subscales, two of which address active and passive function domains. The ArmA ‘active function (AF)’ subscale comprises 13-items (ArmA-AF, score range 0–52) and 8-item ‘passive function (PF)’ subscale (ArmA-PF, score range 0–28). Similarly, the LegA comprises a 15-item active function’ subscale (LegA-AF, score range 0–60) and a 9-item ‘passive function’ subscale (LegA-PF, score range 0–36). Decreasing scores for both measures indicate a reduction in the difficulty of tasks and therefore an improvement in passive or active function. The measures are psychometrically robust, with strong support for construct validity, internal consistency, dimensionality, test–retest reliability, scaling, feasibility, and responsiveness [28,29].

The investigation in this cohort combines arm and leg active and passive function evaluation with practice in a community setting. The importance of longitudinal data to record progress over time and capture real-life clinical decision-making and outcomes was emphasized by the Upper Limb International Spasticity (ULIS) study program [30]. The AboLiSH study [10] provided insights into treatment using BoNT-A for adult leg spasticity. Although these studies examined arm and leg spasticity independently, further work acknowledges that a substantial proportion of individuals experience both concurrently [31].

These large observational studies informed current management of spasticity, representing international clinical practice, diversity of patient presentation, and treatment effectiveness. The authors called for spasticity services to report on their clinical intervention and outcomes to further enhance the evidence base. Some initial ‘real-world’ data has implied that most goals are achieved at similar levels (passive function and pain), but when the primary goal is ‘active function’, this is achieved less frequently due to the complexity involved in the totality of treatment, such as the task training required [32].

This analysis examined outcomes from spasticity management including administration of BoNT-A injections where active and/or passive function of the arm and/or leg was explicitly identified as a treatment goal in routine clinical practice. The service used in this analysis was reflective of the general national model for specialized regional outreach services as described in this paper. The objectives were as follows:

(a) To assess rates of goal attainment for active function and/or passive function in the arm and/or leg spasticity treatment.
(b) To evaluate rates and extent of change (improvement) in active and/or passive function in ArmA for the arm following spasticity treatment.
(c) To evaluate rates and extent of change (improvement) in active and/or passive function in LegA for the leg following spasticity treatment.

## 2. Results

### 2.1. Demographics

From 27 May 2016 to 7 July 2025, 390 patients received spasticity management, including botulinum toxin injection, with a follow-up review of outcomes and completion of a GAS-T score.

The mean age was 60 years (standard deviation 17.1, range 20–99 years). Of the 390 participants, 216 (55.4%) were male, and 174 (44.6%) were female. Table 1 provides the diagnostic etiology of the cohort.

A total of 855 BoNT-A treatment cycles were completed because a proportion of patients received more than one cycle of intervention; see Table 2 for the breakdown per number of cycles. The number of muscles injected per treatment ranged from one (65, 8%) to eight (14, 2%), with the mean number of injections being three.

AbobotulinumtoxinA (Dysport^®^) was administered in 235 treatment episodes, with a mean dose of 706 units (SD 279), range 150–2000 units. IncobotulinumtoxinA (Xeomin^®^) was administered in 620 treatment episodes, with a mean dose of 265 units (SD 121), range 40–1500 units.

Over the course of treatment cycles, in 488 (57%) interventions one goal was set, in 335 (39.2%) interventions two goals were set, in 28 (3.3%) interventions three goals were set, and the remaining 4 had four goals (0.5%) for the intervention.

A total of 1258 individual goals for the treatment of upper and/or lower limb spasticity using botulinum toxin injection were categorized. Overall, 348 (27.7%) goals were set in the domain of symptoms and impairment, whilst 910 (72.3%) goals were related to activities/function. Within the domain of activities/function, 150 (11.9%) were associated with active function, including mobility, and 705 (56.0%) with passive function. Goal categorization is summarized in Table 3.

Injection localization technique was recorded for all interventions, with the use of needle electromyography (EMG)/stimulation (473; 55.3%) being the most frequent, followed by ultrasound (352; 41.2%). Other techniques included anatomical landmark and palpation only (25; 2.9%) or combined EMG/Ultrasound (5; 0.6%).

### 2.2. GAS-T Analysis for Cohort—All Goals

**Figure 1:**
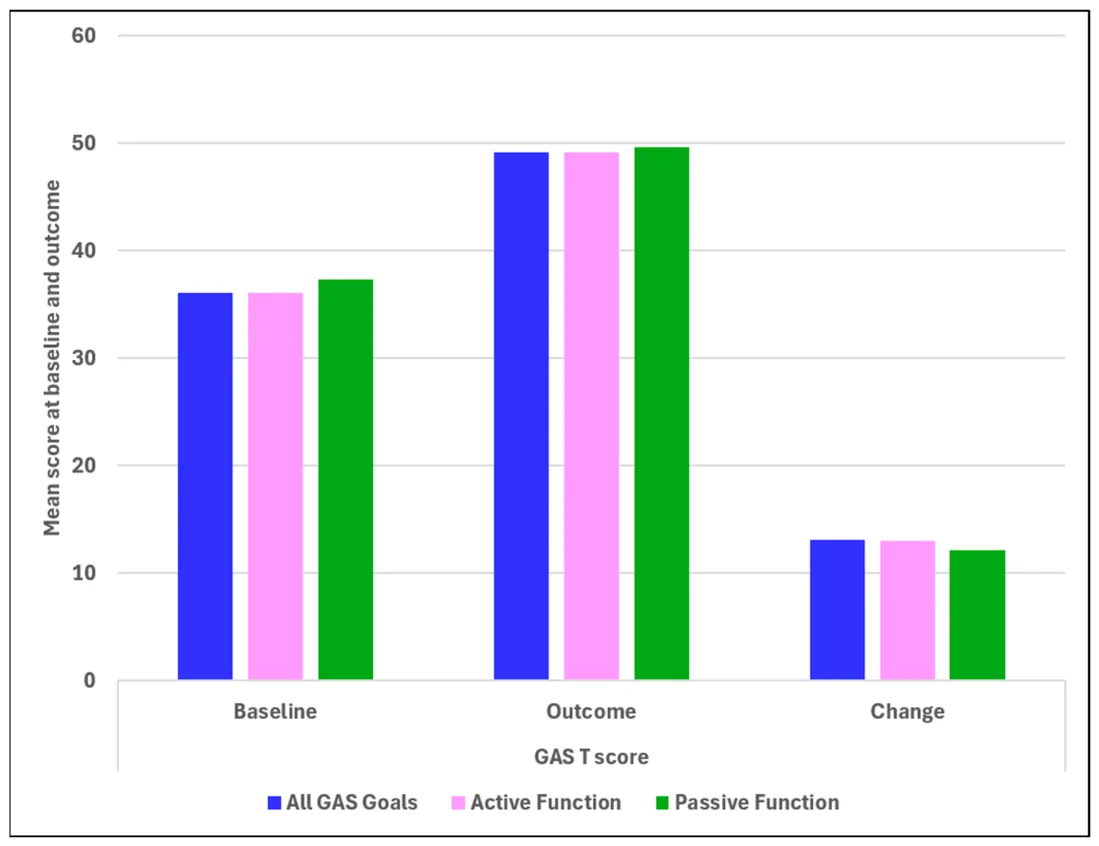
Mean pre- and post-treatment and change GAS-T scores. For GAS-T, higher scores indicate better goal attainment. An increase in GAS-T score reflects improvement following treatment.

The mean (95% CI) GAS-T score at baseline was 36.2 (35.9, 36.5), and the mean GAS-T score at follow-up was 49.4 (48.8, 50.0). The change in GAS-T score was 13.1 (12.5, 13.8), *p* < 0.001. Figure 1 illustrates mean pre- and post- treatment and change GAS-T scores for all goals and for active and passive function goals.

### 2.3. Passive Function

#### 2.3.1. GAS-T

For goals relating to passive function, the mean GAS-T score at baseline was 35.6 (35.2, 36.0), and follow-up was 49.3 (48.6, 50.1). The change in GAS score was 13.7 (12.9, 14.5), *p* < 0.001.

#### 2.3.2. Upper Limb ArmA Passive Function

The mean (95% CI) total ArmA PF score at baseline was 13.9 (13.4, 14.4), and at follow-up 11.4 (10.9, 11.9). The mean change was 2.5 (2.2, 2.8), *p* < 0.001.

#### 2.3.3. Lower Limb LegA Passive Function

The mean total LegA PF score was 14.4 (13.3, 15.4) at baseline and 12.3 (11.3, 13.3) at follow-up. The change was 2.1 (1.5, 2.6), *p *< 0.001.

### 2.4. Active Function

#### 2.4.1. GAS-T

For goals relating to active function, the mean GAS-T score at baseline was 36.3 (35.6, 37.0) and at follow-up was 48.9 (47.3, 50.5). The change in GAS score was 12.6 (10.9, 14.3) *p* < 0.001.

#### 2.4.2. Upper Limb ArmA Active Function

The mean total ArmA AF score at baseline was 40.6. (37.7, 43.4), and at follow-up was 40.1 (37.2, 43.1). The change was 0.4 (−1.4, 2.3), *p* = 0.0.32.

#### 2.4.3. Lower Limb LegA Active Function

The mean total LegA AF score was 28.8 (24.7, 33.0) at baseline, and 27.5 (23.4, 31.6) at follow-up. The change was 1.3 (−0.86, 3.5), *p* = 0.116.

The possibility of multiple testing was minimized, but where the possibility of this occurred, *p*-values were adjusted using the Holm Bonferroni method on a case-by-case basis for each analysis.

**Figure 2:**
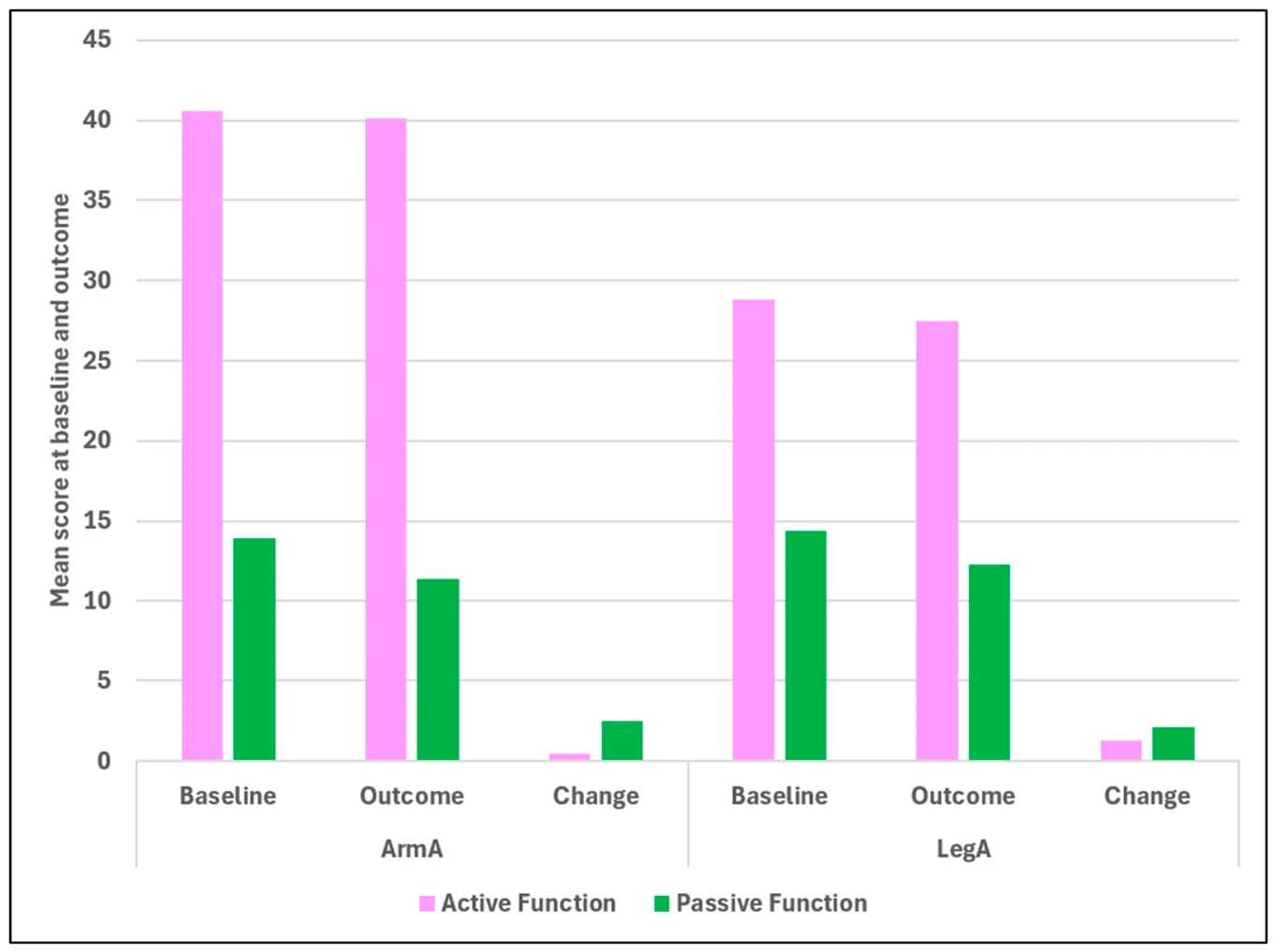
Mean pre-, post-treatment and change scores for ArmA-AF, ArmA-PF, LegA-AF and LegA-PF. For the ArmA and LegA measures, higher scores represent greater activity limitation (worse function), while lower scores indicate improved function.

See Figure 2 for passive and active function ArmA and LegA baseline, outcome and change scores.

### 2.5. Minimal Clinically Important Difference

A clinically significant change was identified for passive and active GAS, with change scores for both greater than 10 (Minimal Clinically Important Difference, MCID 10 or above). Passive function measured by ArmA and LegA reduced significantly and represented MCID for ArmA-PF (change 2.5; MCID 2.5–3).

Active function subscale scores for both ArmA and LegA were both reduced, though this was not significant.

## 3. Discussion

This prospective analysis of goals and standardized outcome measures following BoNT-A injections in a real-world, community-based clinical practice demonstrates improvements in both active and passive upper and lower limb goals, as assessed by GAS. Consistent with these findings, significant improvements were observed in passive function on ArmA and LegA. However, the standardized subscales for active function did not show significant change in this community-based cohort.

### 3.1. Goal Categorization for Active and Passive Function

The prevalence of goal categories from our data is comparable to the original classification publications, with passive function, pain/discomfort, and active function being the most frequent categories for both upper and lower limb [25,33]. Improving passive function is a priority for patients receiving BoNT-A injections, particularly those with more severe disability in the community. This is consistent with findings from previous studies [9]. Identified goals, which are priorities for patients and carers, provide a clear starting point for targeting treatment interventions. The integration of goal setting into clinical routines, with guidance for staff and time resources in place, has been shown to be key to patient-centered goal setting [34]. Our analysis supports the premise that the approach of categorizing goals using a structured framework alongside standardized measures is both feasible and readily applicable within routine clinical practice and is well suited to community-based service delivery.

Many individuals with spasticity present with involvement of both the upper and lower limbs, making a combined intervention and evaluation relevant to both practice and research. While separating arm and leg spasticity can be useful considering the differences in limb function, outcome measures, botulinum toxin dosing, and licensing considerations, analyzing the limbs in isolation may not fully capture the overall impact of spasticity on the individual. An isolated approach risks underestimating the cumulative burden of multi-limb involvement and overlooking the interaction between upper and lower limb impairments that together influence daily activities, participation, and quality of life. The ArmA and LegA assess comparable activity-related constructs specific to each limb, allowing parallel evaluation of upper and lower limb difficulties with a standardized measurement tool, while supporting an integrated assessment of the patient’s overall treatment goals and spasticity-related disability.

### 3.2. Clinical Significance of Outcomes and Implications for Rehabilitation Programs

The clinical and meaningful impact is of primary importance with these clinical interventions to better manage spasticity. GAS (MCID) is arguably not considered in the same manner as standardized measures because a T-score of 50 (or a change of 10 or more) indicates clinical relevance and importance to the individual [21]. The ArmA and LegA are responsive to change, with estimates of clinically meaningful change for the passive subscale(s): 2.5–3 improvement; active subscale(s): 1.1–2.5 improvement.

In this cohort, although GAS demonstrated clinically meaningful improvement in active function goals in this context (change of 12.6 and above the threshold of 10), GAS captures attainment of patient-specific objectives, whereas the ArmA and LegA active-function subscales evaluate performance across a predefined range of items measuring activity. As a result, meaningful gains in targeted functional tasks may not necessarily translate into detectable changes on standardized active-function measures. The smaller changes observed in active function (upper limb 0.4, lower limb 1.3) outcomes may be explained by the fact that reduced function is often driven more by underlying muscle weakness than by spasticity itself. In addition, access to or the dosage of physical therapy may have been insufficient to optimize interventions such as strengthening and task-specific training. Furthermore, the time interval between treatment and follow-up may be insufficient to detect meaningful changes in active function, which often depend on longer-term processes such as muscle strengthening, motor relearning, and skill acquisition. The importance of physical therapy provision and the integration of spasticity management within a holistic rehabilitation program have been emphasized in national guidelines and highlighted by clinicians internationally [1,35]. Early treatment planning for both active and passive goals should therefore be embedded within the broader rehabilitation and care context, with services structured and designed to support sustained engagement in longer-term, integrated rehabilitation [36,37,38].

Overall, the findings of the current study align with those reported in larger real-world cohorts. GAS outcomes were similar to those observed in ULIS and AboLiSH, indicating that the benefits achieved in this service are consistent with wider clinical practice. Furthermore, the overall mean GAS improvement of 13.1 exceeded the established MCID and was greater than the 9.9-point improvement reported in AboLiSH, suggesting that clinically meaningful goal attainment was achieved within this cohort despite its smaller sample size.

### 3.3. Real Life Clinical Practice in Community Settings

The community-based nature of this service is an important consideration when interpreting the findings. Individuals with complex neurological disability often face substantial barriers to accessing hospital-based services due to mobility problems, dependence on carers, transport difficulties and long travel distances. Previous studies have identified considerable unmet needs among community-dwelling adults and care home residents, particularly in relation to contracture prevention, limb care and skin integrity [39]. These challenges are especially relevant for those with the most severe disability, who are often least able to attend conventional outpatient appointments. The predominance of passive function goals within this cohort likely reflects the complexity and severity of disability encountered in community practice.

The outreach model described in this study was designed to address these barriers through the provision of specialist assessment and intervention in patients’ homes (including residential care settings) and satellite community clinics. By delivering specialist spasticity management through this model, community outreach services may facilitate more equitable access to treatment, support intervention at an earlier stage and help prevent secondary complications associated with long-term spasticity, such as contracture, pain and difficulties with personal care.

### 3.4. Implementation of Point-of-Care Ultrasound in a Community Outreach Spasticity Service

Clinical practice has increasingly adopted ultrasound guidance for BoNT-A injections, reflecting a wider move towards greater precision and safety in spasticity management [40]. The study site transitioned from predominantly EMG/stimulation guidance to routine use of point-of-care ultrasound (PoCUS) from August 2021. By enabling real-time visualization of target muscles, adjacent anatomical structures, and needle placement, ultrasound can improve injection accuracy and reduce the risk of complications. Importantly, the portability of modern ultrasound devices has facilitated the delivery of specialist spasticity interventions within community settings.

This study was not designed to compare outcomes before and after PoCUS implementation, nor to evaluate the relative effectiveness of different localisation methods. Therefore, no conclusions can be drawn regarding the direct impact of ultrasound guidance on treatment outcomes within this cohort. Nonetheless, published evidence describing routine use of ultrasound-guided BoNT-A injections in community outreach spasticity services remains limited. As such, the integration of PoCUS into community practice represents an important service innovation, supporting delivery of specialist care beyond traditional hospital environments while maintaining standards of safety and accuracy. Given its operator-dependent nature, implementation requires appropriate governance, competency frameworks, accredited training and ongoing skill maintenance [41,42,43,44]. Specific considerations for upper and lower limb spasticity include recognition of sonographic landmarks, motor-point targeting, identification of adjacent neurovascular structures and safe toxin delivery techniques [45,46,47,48,49,50].

### 3.5. Strengths and Limitations

Key strengths include the large sample size, diverse neurological etiologies, and representation of real-world community practice. The study also demonstrates the practicality of implementing established goal-setting and outcome measurement approaches within a specialist outreach service. As more than one licensed BoNT-A formulation was used during the study period, the findings are likely to be applicable across a range of contemporary spasticity services. However, the analysis was not designed to examine the influence of botulinum toxin formulation, dose, or injection approach on outcomes. Further research is warranted to explore how these factors, including total body dose, are considered when treating both upper and lower limbs, which may influence goal selection and treatment response.

GAS goals and ArmA or LegA active function may evaluate different constructs. Importantly, GAS is always going to evaluate goal attainment, whatever the functional item might be in the context of this evaluation. However, the goal categorization does mean that the broader construction of active function is likely to be maintained (provided our categorization is correct, which we have confirmed in this analysis) and therefore GAS is likely to be more sensitive to change than the standardized measures containing multiple items, some of which may be unlikely to change related to a focal intervention incorporating botulinum toxin administration. This may, in part, explain why change was seen for active function GAS and not for ArmA or LegA active function.

A key limitation of this analysis of routinely collected data is that it is an uncontrolled observational cohort within a multidisciplinary rehabilitation program; causal statements are therefore difficult to make. The results therefore demonstrate associations with goal-directed spasticity management, though from a clinical evaluation of outcome in each case, change and clinical improvement are identified.

Limitations also include the conduct of the study within a single UK specialist service, albeit across multiple community, domiciliary and clinic-based settings. Analyses were limited to individuals with complete baseline and follow-up data; therefore, patients with missing outcome assessments were excluded. These missing data may have introduced selection bias and reduced the available sample size. Additional sources of bias may include variation in clinician expertise, injector proficiency, and treatment approaches across the multidisciplinary teams involved in different locations.

Clinical outcomes in this analysis will have been influenced by differences in the BoNT-A formulation used, the dose of BoNT or physical intervention applied, as well as other anti-spasticity agents, the muscles treated, variability in clinical diagnosis, and the number of treatment cycles applied for the management of a specific goal of intervention. These variables, where possible, have been described and considered in the analysis. However, it was not possible to capture or evaluate all possible variables in this real-life practice analysis. In addition, we acknowledge that some repeated observation occurred for a proportion of the cohort, as detailed in Table 2; however, mixed-effects models were beyond the scope of this paper and were not possible to apply with the current analysis plan and database construction.

## 4. Conclusions

This prospective evaluation of a specialist community spasticity service demonstrates that goal-directed spasticity management, including BoNT-A injection therapy, is associated with clinically meaningful improvements in outcomes that are important to patients and carers. The findings highlight the value of integrating BoNT-A within a broader multidisciplinary rehabilitation framework, recognizing that successful outcomes depend on both focal spasticity management and ongoing rehabilitation interventions.

Findings demonstrate the practicality and clinical utility of combining structured goal setting with standardized outcome measurement across upper and lower limb spasticity in routine community practice. By providing robust real-world evidence from a specialist outreach service, the results support patient-centered goal-focused models of care, particularly in the activities/function domain, for both active and passive function. The results reinforce the role of community-based spasticity services in improving function, reducing care burden, and enhancing everyday participation for people living with complex neurological disability.

## 5. Materials and Methods

### 5.1. Study Design and Participants

Data were prospectively collected at the time of BoNT-A intervention and entered into an electronic database designed for capture of routine data between 2016 and 2025.

The setting was a regional specialized rehabilitation service (United Kingdom—designated Hyper-acute and Level 1 Rehabilitation inpatient unit) with associated outreach and spasticity service.

### 5.2. Inclusion/Exclusion Criteria for This Analysis

**Figure 3:**
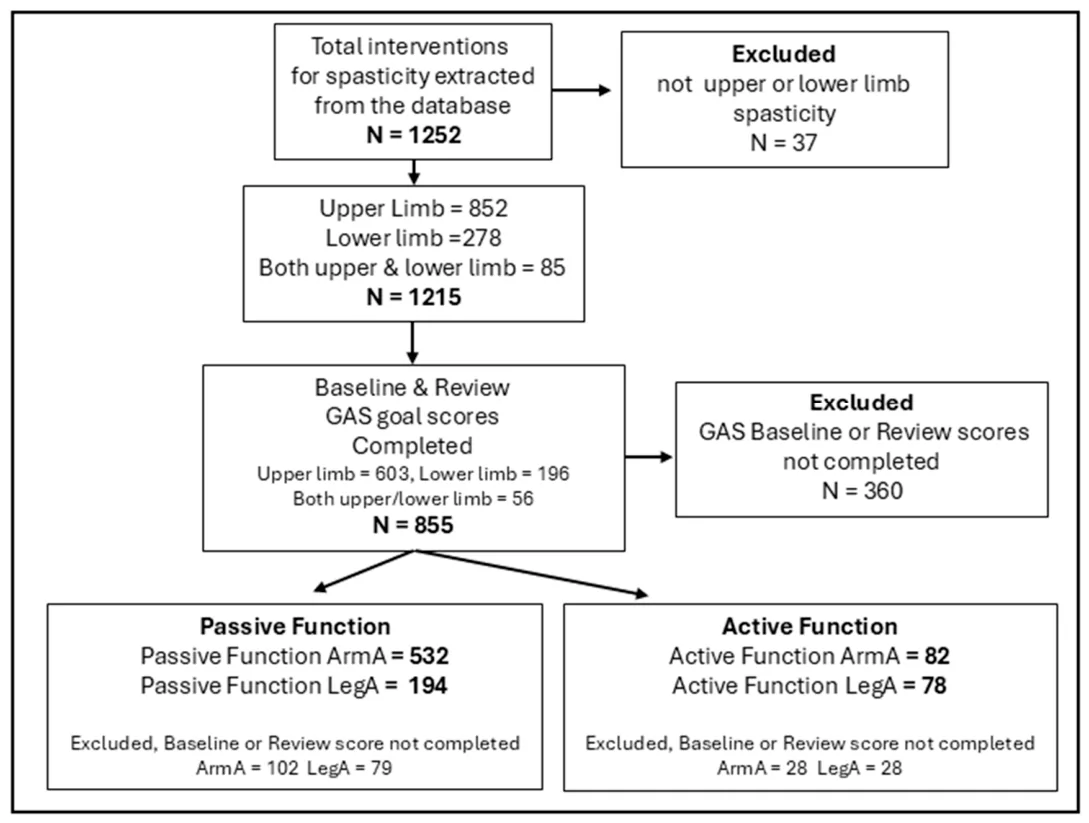
Analysis plan for inclusion and exclusion in alignment with STROBE (Strengthening The Reporting of OBservational studies in Epidemiology).

Patients aged >18 with a neurological diagnosis undergoing spasticity management were included in this analysis if they were entered into the database with Goal Attainment Scale baseline and outcome scores after BoNT-A injection therapy (abobotulinumtoxinA or incobotulinumtoxinA) for focal spasticity of the upper and/or lower limb. Participants were excluded from the data analysis when baseline or review scores of the outcome measures were missing, as illustrated in Figure 3.

### 5.3. Outcome Measures and Assessment

Each patient had one primary treatment goal designated and had the option to identify 2–3 secondary goals that should be amenable to treatment of limb spasticity with BoNT-A. As part of routine assessment and treatment planning, the clinician discussed collaboratively with the patient and/or carer to explore priorities, clarify expectations, and agree on meaningful goals that reflect the patient’s needs and clinical feasibility. All goal statements were to be SMART (Specific, Measurable, Attainable, Realistic and Timely). As part of routine practice, at the time of intervention clinicians categorize goals for treatment using the Goal Attainment Scaling Evaluation of Outcome for Upper Limb Spasticity (GAS-eous) tool [24] or Goal Attainment Scaling for treatment of leg spasticity (GAS-Legs) tool [25], which apply a structured approach to goal-setting and evaluation of goal attainment which an overall GAS T score utilizing the GAS Light method [19].

Baseline GAS scores were derived from baseline goal rating, with goal attainment evaluated at routine follow-up appointments (approximately 6 weeks post-intervention). A GAS T-score is produced using the GAS light method. If goals are achieved as expected, the mean GAS-T score would be 50 (SD +/− 10), with the minimal clinically important change in GAS-T score from baseline reported to be 10 [21].

A standardized outcome measure, ArmA for upper limb and/or LegA for lower limb, was also scored at the time of goal setting. The ArmA is a validated patient-reported outcome measure of perceived difficulty in both passive and active arm function [26,28] and is described in the introduction.

LegA is a patient- or carer-rated measure of difficulty in passive and active leg function, as well as spasticity-related quality-of-life [27,29]. The LegA is a validated patient-reported outcome measure and is described in the introduction.

Both ArmA and LegA are freely available to use and can be obtained with full instructions for completion, from the King’s College London, Department of Palliative Care, Policy and Rehabilitation website: ArmA—Practice resources|Cicely Saunders Institute of Palliative Care, Policy & Rehabilitation|King’s College London and LegA—Practice resources|Cicely Saunders Institute of Palliative Care, Policy & Rehabilitation|King’s College London.

### 5.4. Statistical Analysis

Measures were assessed at baseline and follow-up. Continuous variables are presented as mean values with corresponding 95% confidence intervals (95% CI). Changes between baseline and follow-up were calculated for Goal Attainment Scaling T-scores (GAS-T) for the cohort. The cohort was divided into passive and active sub-cohorts based on GAS goal category. Baseline and follow-up were then evaluated per category for GAS Active Function and Passive Function, Arm Activity Measure Active Function (ArmA-AF) and Passive Function (ArmA-PF), and the Leg Activity Measure Active Function (LegA-AF) and Passive Function (LegA-PF).

Changes from baseline to follow-up were evaluated using paired-sample t-tests. Mean change scores and 95% confidence intervals were calculated to quantify the magnitude and precision of treatment effects. For GAS-T, positive change scores reflect improvement in goal attainment. For ArmA and LegA measures, lower scores indicate reduced impairment; therefore, positive change scores reflect improved function.

To account for possible multiple comparisons due to repeated treatment cycles in a proportion of participants, Holm Bonferroni correction was applied on a case-by-case basis for each analysis. T-tests are reported with mean change (95% CI) and associated *p*-values.

Demographic data are presented descriptively. Continuous variables are summarized as means and standard deviations (mean ± SD), while categorical variables are reported as absolute and relative frequencies, expressed as percentages (%).

In addition, the techniques to guide injections, electromyography, electrical muscle stimulation, and ultrasound were documented.

All analyses were conducted using IBM SPSS Statistics (version 31).

## Figures and Tables

**Table 1:** Etiology of the cohort.

Cohort n = 390	
**Etiology**	**n (%)**
**Acquired Brain Injury**	**334 (85.6%)**
Stroke 236; Trauma 50; Hypoxic 26; Encephalitis 6; Tumor 4; Cerebral Palsy 9; Degenerative (e.g., Vascular Dementia) 3.	
**Progressive neurological condition**	**46 (11.8%)**
Degenerative (e.g., Parkinsons) 37; Inflammatory (e.g., multiple sclerosis) 9.	
**Spinal Cord Injury**	**10 (2.6%)**
Trauma 4; Degenerative 3; Inflammatory 1; Tumor1; Infarct 1.	

Key: Bold indicates category.

**Table 2:** Number of treatment cycles.

Number of Treatment Cycles	Patients (n = 390)
1	230 (59%)
2	68 (17%)
3	32 (8%)
4	18 (5%)
5	14 (4%)
6	8 (2%)
7	8 (2%)
8+	12 (3%)

**Table 3:** Goal categorization.

Total Goals Set n = 1258		
	**Frequency**	**%**
**Domain 1: symptoms/impairment**	**348**	**27.7**
Pain/discomfort	212	16.9
b. Involuntary movement	16	1.3
c. Range of movement/contracture prevention	120	9.5
**Domain 2: Activities/function**	**910**	**72.3**
Passive function (ease of caring for the affected limb)	705	56.0
b. Active Function (including mobility)	150	11.9
c. Facilitating therapy	55	4.4

## Data Availability

The original contributions presented in this study are included in the article. Further inquiries can be directed to the corresponding author.
